# Rationalizing the use of common parameters and technological tools to follow up *Mycoplasma hyopneumoniae* infections in pigs

**DOI:** 10.1186/s40813-024-00381-x

**Published:** 2024-08-23

**Authors:** Karina Sonalio, Filip Boyen, Bert Devriendt, Ilias Chantziaras, Lisa Beuckelaere, Evelien Biebaut, Freddy Haesebrouck, Irene Santamarta, Luís Guilherme de Oliveira, Dominiek Maes

**Affiliations:** 1 Department of Internal Medicine, Reproduction and Population Medicine, Faculty of Veterinary Medicine, Ghent University, Merelbeke, Belgium; 2 Department of Veterinary Clinic and Surgery, School of Agricultural and Veterinarian Sciences, São Paulo State University (Unesp), Jaboticabal, Brazil; 3 Department of Pathobiology, Pharmacology and Zoological Medicine, Faculty of Veterinary Medicine, Ghent University, Merelbeke, Belgium; 4 Department of Translational Physiology, Infectiology and Public Health, Faculty of Veterinary Medicine, Ghent University, Merelbeke, Belgium; 5 Laboratorios Syva SAU, León, Spain

**Keywords:** Movement, Microchip, Sensors, Respiratory disease, Experimental infection, Swine

## Abstract

**Background:**

*Mycoplasma (M.) hyopneumoniae* is associated with respiratory disease in pigs and is the primary agent of enzootic pneumonia. Quantification of *M. hyopneumoniae*-related outcome parameters can be difficult, expensive, and time-consuming, in both research and field settings. In addition to well-established methods, technological tools are becoming available to monitor various aspects of relevant animal- and environment-related features, often in real-time. Therefore, this study aimed to assess whether certain parameters, such as animal movement and body temperature using microchips (IMT), correlate with established parameters and whether the currently used parameters can be rationalized.

**Results:**

The percentage of movement was significantly reduced by *M. hyopneumoniae* infection in pigs (*p* < 0.05), where the *M. hyopneumoniae*-infected group showed a lower percentage of movement (1.9%) when compared to the negative control group (6.9%). On the other hand, macroscopic (MLCL) and microscopic (MLL) lung lesions, respiratory disease score (RDS), *M. hyopneumoniae*-DNA load, and anti-*M. hyopneumoniae* antibody levels increased significantly in the *M. hyopneumoniae*-infected group 28 days post-inoculation (*p* < 0.05). Moderate (*r* > 0.30) to very strong correlations (> 0.80) were observed between the abovementioned parameters (*p* < 0.05), except for IMT. A significant and moderate correlation was reported between IMT and rectal temperature (*r* = 0.49; *p* < 0.05). Last, the average daily weight gain and the percentage of air in the lung were not affected by *M. hyopneumoniae* infection (*p* > 0.05).

**Conclusions:**

*M. hyopneumoniae* infection significantly reduced the movement of piglets and increased lung lesions, *M. hyopneumoniae-*DNA load, and anti-*M. hyopneumoniae* antibody levels; and, good correlations were observed between most parameters, indicating a direct relationship between them. Thus, we suggest that changes in movement might be a reliable indicator of *M. hyopneumoniae* infection in pigs, and that a selected group of parameters—specifically RDS, MLCL, MLL, *M. hyopneumoniae*-DNA load, anti-*M. hyopneumoniae* antibody levels, and movement—are optimal to assess *M. hyopneumoniae* infection under experimental conditions.

**Supplementary Information:**

The online version contains supplementary material available at 10.1186/s40813-024-00381-x.

## Background

Respiratory diseases are a major health challenge in swine production worldwide. This is especially the case for enzootic pneumonia (EP), a chronic respiratory disease, that negatively impacts the health and growth of pigs. *Mycoplasma hyopneumoniae (M. hyopneumoniae)* is the primary pathogen of EP and plays an important role within the Porcine Respiratory Disease Complex (PRDC), together with other pathogens such as porcine circovirus type 2, porcine respiratory reproductive syndrome virus (PRRSV), and *Pasteurella multocida* [1].

*M. hyopneumoniae* colonizes the mucosal surface of the trachea, bronchi, and bronchioles, affecting the mucosal clearance system by breaking the cilia and modulating the respiratory tract immune system [2, 3]. Strains of *M. hyopneumoniae* have been described to be antigenically and genetically diverse [2, 4–6], and differences in virulence have been reported, with high virulence strains inducing more severe pneumonia in a greater proportion of infected pigs [7–11]. This might be attributed to a greater capacity of the bacteria to multiply in the bronchioles, resulting in a more severe inflammation [11]. Additionally, the pathogenesis of *M. hyopneumoniae* is complex and involves long-term colonization of the ciliated epithelium, stimulation of a prolonged inflammatory reaction, modulation of innate and adaptive immune responses, and interaction with other infectious agents [2].

Although an acute inflammatory response is triggered by *M. hyopneumoniae* infection in the lungs, the disease predominantly occurs as a chronic condition, in which the unproductive cough is the most prominent clinical sign [2, 7]. In addition to that, shortness of breath, dyspnea, and fever may occur upon infection [2]. Macroscopic lung lesions can be found in the apical, cardiac, and accessory lobes and, sometimes, in the cranioventral part of the diaphragmatic lobes [2]. These lesions consist of consolidated areas that range from purple to gray [12]. At the microscopic level, *M. hyopneumoniae* triggers the development of broncho-interstitial pneumonia, initiating with limited neutrophil accumulation and progressing to marked accumulation of neutrophils, fluid, and macrophages in alveoli. This leads to the proliferation of type II alveolar pneumocytes, increased lymphocyte, histiocyte, and plasma cell accumulation, peribronchial lymphoid nodules, and subsequent development of alveolar pneumonia. The final stages involve peribronchial lymphoid hyperplasia, increased perivascular mononuclear cells, and extensive peribronchial cuffs with numerous lymphocytes and fewer plasma cells, forming large compressive lymphoid nodules [2, 13, 14].

Diagnostics of *M. hyopneumoniae* infection comprehend the evaluation of non-pathognomonic parameters, such as clinical signs (cough, fever), macroscopic and microscopic lung lesions, average daily weight gain, percentage of air in the lung, and specific diagnostic tools for direct or indirect detection of *M. hyopneumoniae*, for example, using PCR [15–17] and ELISA tests [2]. Although commonly used, evaluating these parameters might be labor-intensive, time-consuming, and expensive [18], raising questions on the added value of some parameters [19]. Furthermore, the meticulous selection of the most relevant parameters to be evaluated in *M. hyopneumoniae*-related research would enable us to optimize diagnostic accuracy significantly, thereby maximizing the efficiency of the allocated resources [19, 20].

Since there is an increasing need for real-time monitoring of health and performance parameters in pig farming to increase productivity and sustainability, recent technologies like intelligent sensors (cameras, microphones, and microchips) and artificial intelligence are being introduced [21]. Moreover, these technologies can be easily applied in experimental settings to optimize challenge models, efficacy studies, and clinical diagnosis [2, 21]. An interesting parameter to monitor is the movement of animals, as changes in the frequency of movement have been shown to be a key indicator of health or welfare problems and to assist in early disease detection [22]. In addition, in early infections, one of the first physiological changes is often the increase in body temperature, and thus, being able to assess body temperature without placing the pigs under unnecessary stress e.g., by using thermal microchips or infrared cameras, can be valuable [23, 24]. Although the movement behavior of pigs has been assessed for some respiratory diseases, such as Porcine Reproductive and Respiratory Syndrome (PRRS) and porcine pleuropneumonia (*Actinobacillus pleuropneumoniae*), it has not yet been investigated for *M. hyopneumoniae* infection. Likewise, changes in intramuscular temperature (IMT) have not yet been monitored during *M. hyopneumoniae* infection. Therefore, the aims of the present study were: (i) to assess the impact of *M. hyopneumoniae* infection on the movement and the IMT of piglets by using a camera monitor and a thermal microchip, respectively; (ii) to investigate the correlations between movement, IMT, and the conventionally used parameters, such as respiratory disease score, macroscopic and microscopic lung lesions, percentage of air, average daily weight gain, *M. hyopneumoniae-*DNA load, and anti-*M. hyopneumoniae* antibody levels.

## Results

Six animals (two from each infected group) died during or shortly after the inoculation on D0 or D1, and therefore, they were excluded from all analyses. No specific lesions were observed during the necropsy, indicating that the death of those piglets was probably associated with the stress of the manipulation or other factors intrinsic to the individuals. At euthanasia, mild pleurisy and/or small abscesses were observed in 10 piglets, of which five were from the negative control (NC) group, two from S1, and three from S3. Mild clinical signs of post-weaning diarrhea were observed in all groups from D-6 onwards and were resolved by D-1, after individual treatment with colistin.

In the BALF (D28) of two animals (from NC and S3) *Trueperella pyogenes* and *Pasteurella multocida* were identified. All animals from the control group remained negative for *M. hyopneumoniae*-specific antibodies and DNA throughout the experimental period.

### Assessment of movement, sound, and environmental conditions

**Fig. 1:**
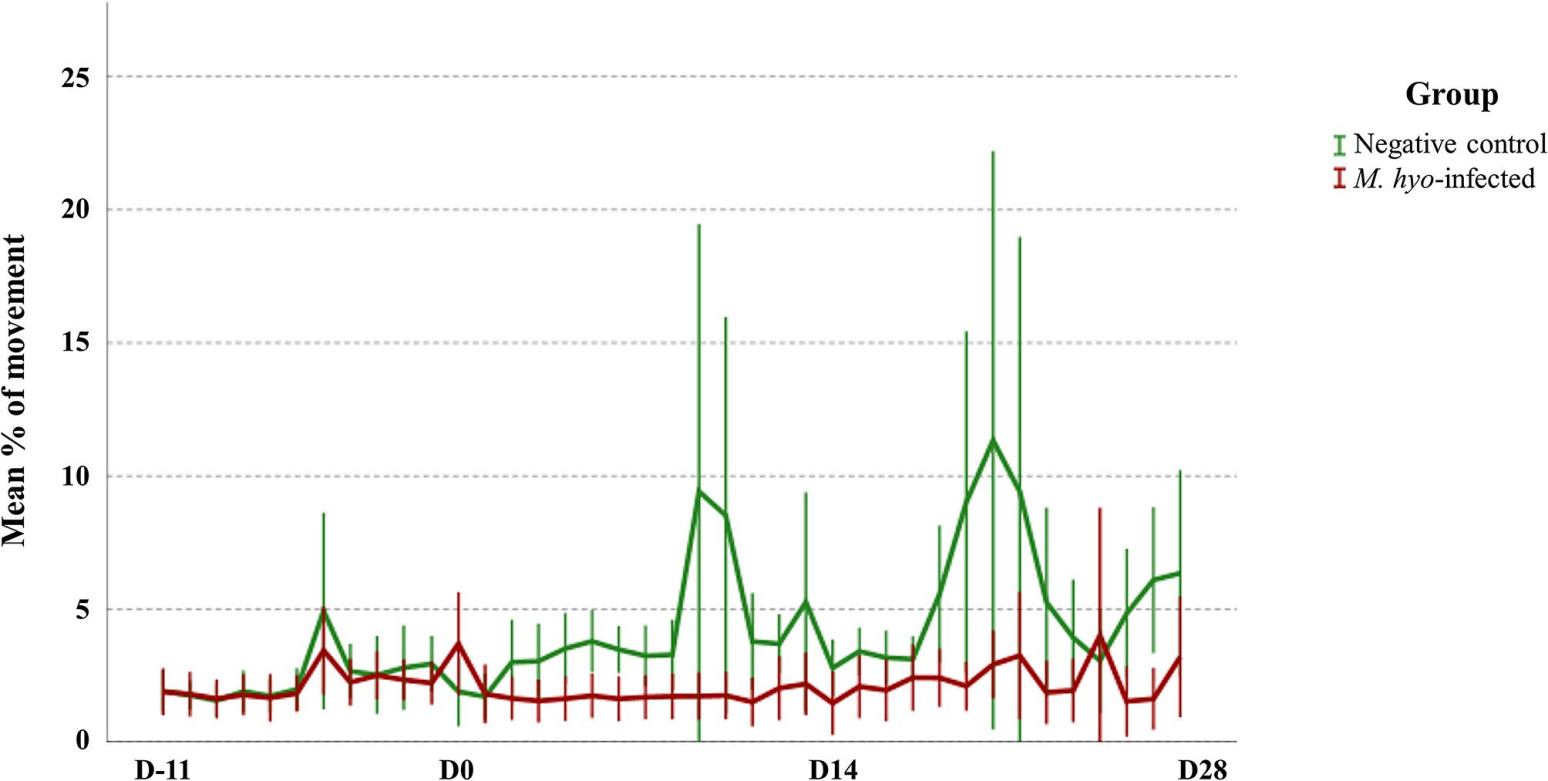
Mean percentage of movement throughout the study period. D0 refers to the inoculation day. Error bars represent the confidence interval of 95%

During the post-inoculation period (D0 to D28), the *M. hyopneumoniae*-infected group showed a lower percentage of movement (1.9%) when compared to the negative control group (6.9%; *p* < 0.05) (Fig. 1), and the odds of movement after inoculation in the NC group was 28.68 times greater than in the *M. hyopneumoniae* group (*p* < 0.001). Prior to the inoculation, the average percentage of movement was 1.6% for all groups, whereas upon inoculation with the *M. hyopneumoniae* strains, lower values were observed in the individual infected groups (S1, S2, and S3) when compared to the NC (Additional file 1). Furthermore, the group factor (infected or non-infected) negatively affected the movement (*p* < 0.001), while the factor inoculation (pre- or post-inoculation) showed a tendency to decrease the movement (*p* = 0.054).

Concerning the environmental parameters, similar results were observed among the groups, except for dust particles, for which higher values were observed in the NC (Additional file 2). Similarly, higher decibel values for the 602 Hz frequency were observed in the control group compared to the infected groups (Additional files 2 and 3).

### Evaluation of clinical signs and weight gain

Concerning the RDS data, coughing was first reported on D3 in the *M. hyopneumoniae* group and increased progressively until D28, as shown in Additional file 4 A. Similarly, a progressive increase in coughing was observed for the individual groups, except for the NC group (Addition file 4B). The RDS post-inoculation was significantly higher in the *M. hyopneumoniae-*infected groups compared to the NC group (*p* < 0.05). Lastly, there were no significant differences in the ADWG between the groups (Table 1).

**Table 1:** Mean (M) and standard deviation (SD) values of the different parameters assessed in the negative control group and *M. hyopneumoniae*-infected group. Within the same row, values with different letters are significantly different *(p ≤* 0.05*)*

Parameter	Study period	Negative control group		M. hyopneumoniae-infected group	
		M	SD	M	SD
*RDS post-inoculation*	0 to 28	0.06 _a_	0.20	0.97 _b_	0.83
*IMT post-inoculation*	0 to 28	39.59 _a_	0.20	39.70 _a_	0.20
*ADWG in g/pig/day*	-12 to 0	113.75 _a_	48.00	113.56 _a_	47.67
	-12 to 28	327.69 _a_	90.00	362.11 _a_	83.00
	0 to 28	419.38 _a_	124.00	468.63 _a_	110.00
*Anti-*M. hyopneumoniae *antibody levels*	28	0.00 _a_	0.00	0.67 _b_	0.27
M. hyopneumoniae*-DNA load (Log10 copies/µL)*	14	0.00 _a_	0.00	3.85 _b_	0.47
	28	0.00 _a_	0.00	4.43 _b_	0.33
*MLCL score*	28	0.57 _a_	1.30	4.33 _b_	2.40
*MLL score*	28	2.16 _a_	0.20	2.63 _b_	0.33
*% of air in lung slides*	28	37.33 _a_	4.50	34.56 _a_	6.13

RDS: respiratory disease score; IMT: intramuscular temperature (°C); ADWG: average daily weight gain; MLCL: macroscopic lung consolidated lesion; and MLL: microscopic lung lesion

### Assessment of body temperature

**Fig. 2:**
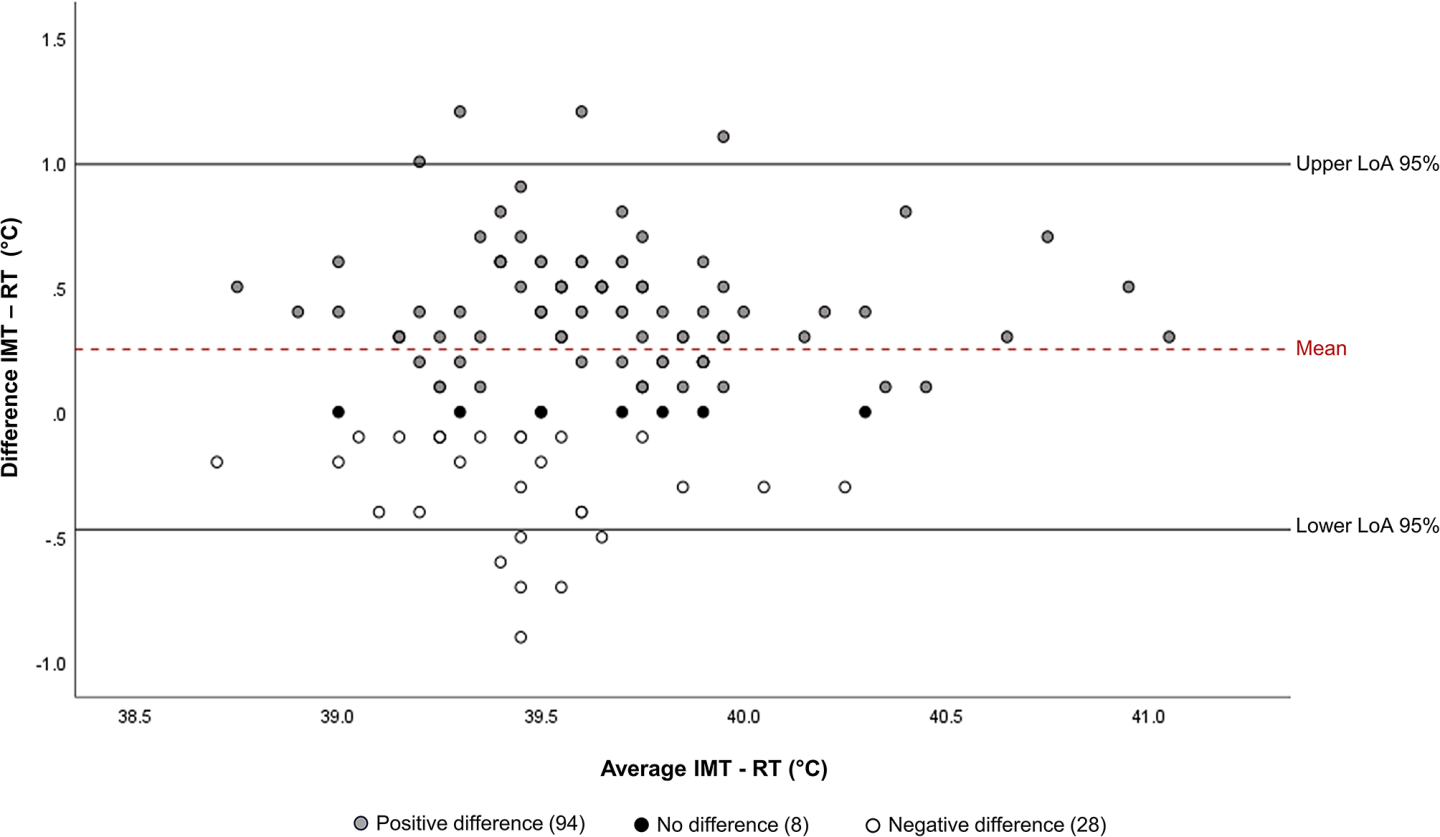
Bland-Altman plot showing the level of agreement (LoA) between intramuscular temperature (IMT) and rectal temperature (RT) measurements, in a 95% confidence interval (CI)

Although slightly higher values were reported in the *M. hyopneumoniae*-infected group, we did not see a significant difference in IMT of pigs upon *M. hyopneumoniae*-infection (*p* > 0.05). Likewise, no differences were seen between the individual groups, where values ranged from 38.4 °C to 41.3 °C (Additional file 5). Variation was 0.21 for the IMT and 0.18 for the RT. A mean difference of 0.25 °C (95% CI = 0.20–0.35) was observed (*p* < 0.001) between IMT and RT and in most cases (94/130), IMT was higher, as shown in Fig. 2. There was a significant moderate correlation (*r* = 0.53; *p* < 0.001) between IMT and RT (Table 2). Lastly, IMT post-infection did not differ significantly between *M. hyopneumoniae*-infected and NC groups, although the average values for the *M. hyopneumoniae*-infected group were slightly higher (+ 0.11 °C; Additional file 6).

### Macroscopic and microscopic lung lesion score

Small *Mycoplasma*-like lung lesions were observed in two piglets from the NC group at necropsy (D28), even though these piglets tested *M. hyopneumoniae-*negative by dPCR (D14 and D28) and did not seroconvert (D0 and D28). All piglets in the different infected groups (S1, S2, and S3) developed *Mycoplasma-*like lung lesions by D28. The mean MLCL and MLL scores were significantly higher in the *M. hyopneumoniae* group in comparison to the NC group *(p <* 0.05*)*, while the percentage of air in the lung tissue was not statistically different between both groups (Table 1). The average values for MLCL and MLL scores, the percentage of air, and other parameters of the groups NC, S1, S2, and S3 are shown in Additional file 7.

### Detection of M. hyopneumoniae DNA in BALF and specific antibodies in serum

All control animals tested negative for *M. hyopneumoniae* DNA (D14 and D28), while all infected animals were positive (Additional file 7) and an increase in the DNA load was detected from D14 to D28 (*p <* 0.05). All animals were seronegative on D0. The control animals remained negative until D28, while the *M. hyopneumoniae*-infected animals seroconverted by D28, except for one animal from the S2 group and one from the S3 group. Additionally, a statistically significant difference *(p <* 0.05*)* between NC and the *M. hyopneumoniae* group was reported for *M. hyopneumoniae*-DNA load (D14 and D28) and anti-*M. hyopneumoniae* antibody levels (D28), as shown in Table 1.

### Associations between the evaluated parameters

Significant correlations were reported among most parameters assessed in this study, as shown in Table 2. A very strong (*r* > 0.80) negative correlation was observed between MLL and percentage of air, whereas strong positive correlations (*r* = 0.60 to 0.80) were present between MLCL, RDS post-inoculation, *M. hyopneumoniae*-DNA load, and anti-*M. hyopneumoniae* antibody levels. Positive and moderate correlations (*r* = 0.30 to 0.59) were found between MLCL, MLL, *M. hyopneumoniae*-DNA load, RDS post-inoculation, and anti-*M. hyopneumoniae* antibody levels. Lastly, no significant correlations were observed for the IMT post-inoculation data and the other clinical parameters, besides a moderate correlation between IMT and RT (*r* = 0.49; *p* > 0.05).

**Table 2:**
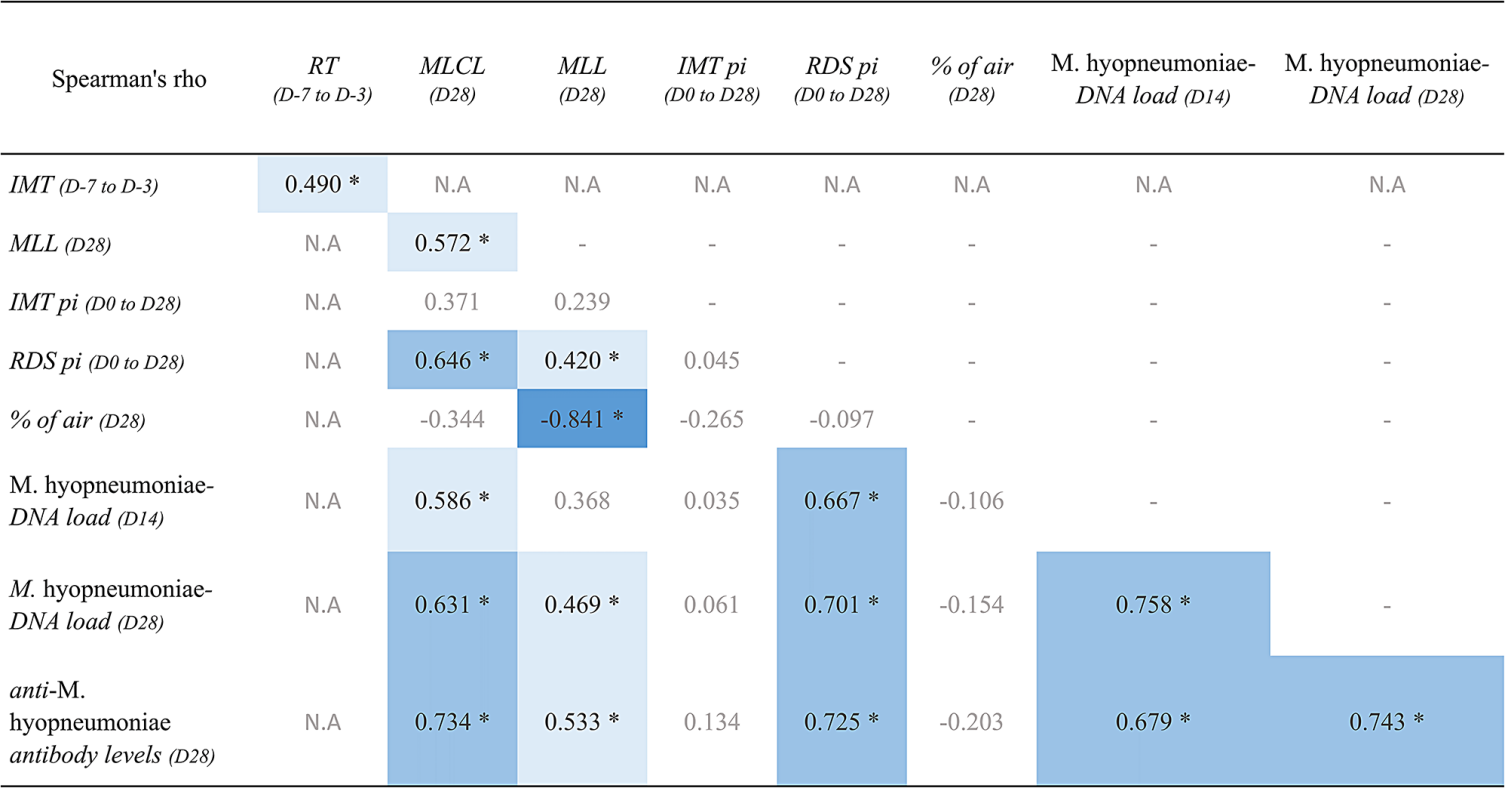
Spearman’s correlation coefficient matrix of the main clinical parameters. RT: rectal temperature; MLCL: macroscopic lung consolidated lesion; MLL: microscopic lung lesion; IMT Pi: intramuscular temperature post-inoculation (D0 to D28); RDS pi: respiratory disease score post-inoculation (D0 to D28); D: day

## Discussion

The present study showed that *M. hyopneumoniae* infection reduced movement and, as expected, increased MLCL, MLL, RDS, *M. hyopneumoniae*-DNA load, and anti-*M. hyopneumoniae* antibody levels. Percentage of air, ADWG, and IMT were not affected. It was also shown that intramuscular thermochips can be an alternative tool to assess body temperature under experimental conditions. Furthermore, contemporary *M. hyopneumoniae* isolates (11.1 A and 13.1B) showed similar results on the main clinical parameters, such as MLCL, MLL, RDS, *M. hyopneumoniae*-DNA load, and anti-*M. hyopneumoniae* antibody levels, to those observed with the reference *M. hyopneumoniae* strain F7.2 C (S1).

Most infectious diseases can decrease the activity and movement behavior of animals [25–27], making this information useful for welfare monitoring and early disease detection. In this study, changes in the movement were seen from D2 onwards, preceding the changes in the RDS (D8 onwards). In addition, we observed a lower percentage of movement in the *M. hyopneumoniae*-infected group and a higher chance of movement in the control group, confirming the hypothesis that *M. hyopneumoniae* infection negatively influences the movement of pigs. Similar results were reported for PRRSV infection in pigs. The piglets in the PRRS-positive group moved less, and also spent less time feeding when compared to the control group [28]. However, in that previous study, both PRRS-negative and PRRS-positive animals were also infected with *M. hyopneumoniae*, indicating that PRRSV infection further decreased movement in *M. hyopneumoniae-*infected animals. It is believed that, in the present study, the infectious agent (*M. hyopneumoniae*) was the main pathogen affecting the pigs’ movement as the animals originated from the same specific pathogen free-herd and strict biosecurity measures were applied between the rooms, throughout the entire study period. Nonetheless, investigating the movement of *M. hyopneumoniae*-positive and negative herds or batches would be essential for a comprehensive understanding of the movement behavior of *M. hyopneumoniae*-infected and -non-infected pigs under field conditions.

The activity and movement patterns of pigs can be influenced by various factors, such as the space allocated per animal, housing conditions (including floor type, temperature, and access to toys), as well as age and weight [29–31]. Studies have indicated a decline in walking and standing behavior with age, primarily attributed to the limited space available per pig, a factor directly impacted by the pig’s growth [30, 31]. In our study, the piglets were kept in separate, but very similar rooms (same size, floor, heating, same number and location of toys and feeders), and with the same feed and water management. Interestingly, in our study, the movement of piglets appeared not to be affected by age or space allocation per pig, possibly due to the relatively brief duration of our observation period (seven weeks) and the available floor area per pig.

In our study, piglet movement behavior was negatively affected by *M. hyopneumoniae* infection, suggesting that this parameter could be used for respiratory disease detection in a more chronic setting alongside well-established parameters such as MLCL, RDS, *M. hyopneumoniae-*DNA load, and *M. hyopneumoniae*-specific antibodies. The main advantage of such a system is that it can then be done in an objective and quantitative way. Besides, the continuous assessment of movement on farms could also be highly valuable to veterinarians, serving as a potential tool for monitoring and managing pig herd health, enabling early disease outbreak detection. On top of that, if algorithms had the capacity to discriminate between animals infected with specific pathogens, such as *M. hyopneumoniae* and PRRSV, by analyzing changes in movement, it might transform the landscape of personalized medicine within agricultural contexts, leading to groundbreaking progress. Even though our results encourage further research into this topic, changes in movement alone should be interpreted with caution for diagnostic purposes, especially because it can be influenced by many factors, not only by infections with pathogens.

Environmental conditions, such as high levels of ammonia, carbon dioxide, and dust particles, and deviations from normal temperature, humidity, and ventilation rate are known risk factors for developing and aggravating respiratory diseases in pigs [2, 32], and they may also influence the level of movement activity [33]. In this study, these conditions were similar between the groups, except for the dust particles (10 µg/m^3^) and sound levels at 602 Hz. Higher values were observed for the NC group compared to the infected groups. The greater values of dust particles observed in the NC group may be related to the movement activity of the pigs, although other factors such as an increased feed spill could also have played a role. Regarding the sound level data, numerical differences were observed within the 602 Hz frequency, where animals in the NC group produced more intense sounds throughout the study when compared to the infected groups. Although lower sound frequencies (400–600 Hz) have been associated with coughing caused by infectious agents, such as *Pasteurella multocida* [34], the numerical difference observed in the 602 Hz frequency between NC and *M. hyopneumoniae-*infected groups should be interpreted with caution, especially because the difference was already reported before the inoculation of the animals, indicating that other factors such as housing conditions or malfunctioning of the sound sensors could have played a role.

The main clinical sign of *M. hyopneumoniae* infection in pigs is non-productive coughing. However, body temperature [35] and performance parameters, such as ADWG, can also be affected [36]. The onset of the coughing starts between one and two weeks after infection and increases up to five weeks [2]. Not surprisingly, RDS data were significantly higher in the infected animals from 10 days post-inoculation (dpi) to 28 dpi as compared to the control animals, corroborating previous studies [7, 37–39]. These increased RDS values can be directly associated with the development of the disease and its inflammatory process [2]. This is supported by the strong correlations between RDS post-inoculation on the one hand and MLCL, *M. hyopneumoniae*-DNA load, and anti-*M. hyopneumoniae* antibody levels on the other hand. Similar results were reported when different *M. hyopneumoniae* strains and challenge models were used [7, 35]. Concerning ADWG, no differences were observed between the groups in the different periods in our study, which is likely due to the rather short study duration and the limited number of animals used [7].

Assessment of the RT in pigs is often used in experimental trials but it is not very convenient when repetitive measurements must be taken. In addition, measuring RT may not reflect the temperature at rest, as it might increase due to the movement and possible stress of handling. This might also result in a high variation of temperature data between pigs. Thus, alternative methods, such as thermal transponders and portable readers, gain increasing interest [40–42]. Different temperature results were reported when microchips were placed in different places of the body, such as subcutaneous, intramuscular, or intraperitoneal implantation [23, 40–43]. In our study with intramuscularly implanted microchips, a good correlation between IMT and RT was observed, and median IMT values were 0.25 °C higher than the corresponding median RT values. Previous studies also reported a correlation between RT and IMT, although the average IMT values were slightly lower than the RT values [40, 43]. This could have been attributed to an increase in temperature just before RT measurement (possibly due to handling and stress), and also to the location of the microchip, as microchips implanted subcutaneously lead to lower values compared to RT data [41]. The IMT did not differ significantly between *M. hyopneumoniae*-infected and NC groups. This might be due to the low sample size and measurement frequency. Nonetheless, considering the ease of use and good correlation with RT, thermal microchips seem to be a convenient and accurate alternative to assess the body temperature of pigs under experimental conditions.

Although not pathognomonic, cranioventral pulmonary consolidations in the lung are suggestive of *M. hyopneumoniae* infection in pigs [2]. Likewise, broncho-interstitial pneumonia with hyperplasia of bronchus-associated lymphoid tissue lesions is characteristic of *M. hyopneumoniae* infection. In our study, macroscopic and microscopic lesions differed significantly between the infected and control animals. The MLCL were moderately to strongly associated with MLL, *M. hyopneumoniae*-DNA load, and anti-*M. hyopneumoniae* antibody levels. A very strong and negative correlation between MLL and the percentage of air was reported in our study, which is logical as the presence of lymphocytes, macrophages, and neutrophils increases with stronger inflammatory responses, and consequently decreases the percentage of air in the alveoli [2, 11, 17]. Despite, the strong correlation, no significant differences were observed for the percentage of air between the NC and *M. hyopneumoniae*-infected groups, which could be due to the great variability of the data. Thus, as MLL holds more intrinsic and direct information concerning the degree of inflammation when compared to the percentage of air, we prefer the assessment of MLL over assessing the percentage of air in the lung tissue.

Lastly, regarding the production of *M. hyopneumoniae*-specific antibodies upon *M. hyopneumoniae* infection, strong correlations between anti-*M. hyopneumoniae* antibody levels and the key clinical parameters (MLCL, MLL, RDS, and *M. hyopneumoniae*-DNA load) were observed. Most of the infected animals (16/18) seroconverted by D28. Previous studies reported higher numbers of piglets seroconverting by 28 dpi when using highly virulent strains compared to low virulent strains [7, 11]. In addition, based on the similar results described for the infected groups, we believe that these contemporary *M. hyopneumoniae* isolates (strain 11.1 A and 13.1B) might have a virulence similar to the F7.2 C strain. However, since the piglets were also challenged with a low virulent strain (F1.12 A), further research is needed to assess the virulence of the new isolates in the absence of a subsequent challenge.

Whereas our findings are important and can be useful to monitor *M. hyopneumoniae* infection in pigs, some limitations should be mentioned. First, the study was done under experimental conditions with a limited number of animals and a short duration. Therefore, the results do not reflect the field situation, where animals are kept for a longer period, are subjected to different housing and management conditions, and have different sanitary challenges. These conditions could also influence the movement of pigs, making it more difficult to associate the change in movement with a specific pathogenic infection. Furthermore, the use of thermal microchips to assess body temperature in the field is currently seldom used, possibly due to the risk of becoming a hazard in the meat, as locating it during the slaughter process would not be feasible. Lastly, although no added value regarding weight gain was observed in our study, ADWG remains of great relevance, particularly for long-term studies, field experiments, and the swine industry in general.

Therefore, considering the results, a specific set of parameters such as movement, macroscopic and microscopic lung lesion scores, coughing (RDS), *M. hyopneumoniae-DNA load*, and anti-*M. hyopneumoniae* antibody levels can be used to efficiently monitor experimental *M. hyopneumoniae* infection in pigs. The percentage of air in the lung and ADWG had no added value, especially for rather short studies. Thus, the present study provides critical insights into which parameters could be evaluated upon *M. hyopneumoniae* infection to reach a maximum efficiency of used resources under experimental conditions. Further research is nevertheless needed to investigate the outcome of these parameters in different study conditions.

## Conclusions

The present study showed that the movement of pigs was negatively affected by experimental infection with *M. hyopneumoniae*. Body temperature data assessed using microchips was on average 0.25 °C higher than but correlated well with data from rectal temperature measurements. Moderate to strong correlations were observed between all assessed parameters, except IMT. Based on our findings, we suggest the use of movement data for early disease detection, followed by the comprehensive evaluation of a specific set of parameters—specifically macroscopic and microscopic lung lesion scores, coughing (RDS), *M. hyopneumoniae-*DNA load, and anti-*M. hyopneumoniae* antibody levels—to assess *M. hyopneumoniae* infection in pigs.

## Methods

### Experimental design

This study was approved by the Ethics Committee of the Faculty of Veterinary Medicine and the Faculty of Bioscience Engineering at Ghent University (EC 2021-062), and complies with European Directive 2010/63/EU.

Thirty-two, 28-day-old piglets (16 males and 16 females; Naima x Piétrain), were purchased from a *M. hyopneumoniae*-free herd, which is a farrow-to-finish farm with 300 sows and producing according to a three-week system. The herd has previously tested seronegative and PCR-negative for *M. hyopneumoniae* [17], and no *Mycoplasma hyopneumoniae*-like lung lesions were reported during the last four years. One month before the start of the trial, tracheobronchial swabs were collected from 50 pigs of five different age groups (7–8 weeks, 11 weeks, 16 weeks, 22 weeks, and 26 weeks) and tested by nPCR for the presence of *M. hyopneumoniae* DNA [44]. All samples tested negative. The farm was also free from PRRSV and *Actinobacillus pleuropneumoniae.*

**Fig. 3:**
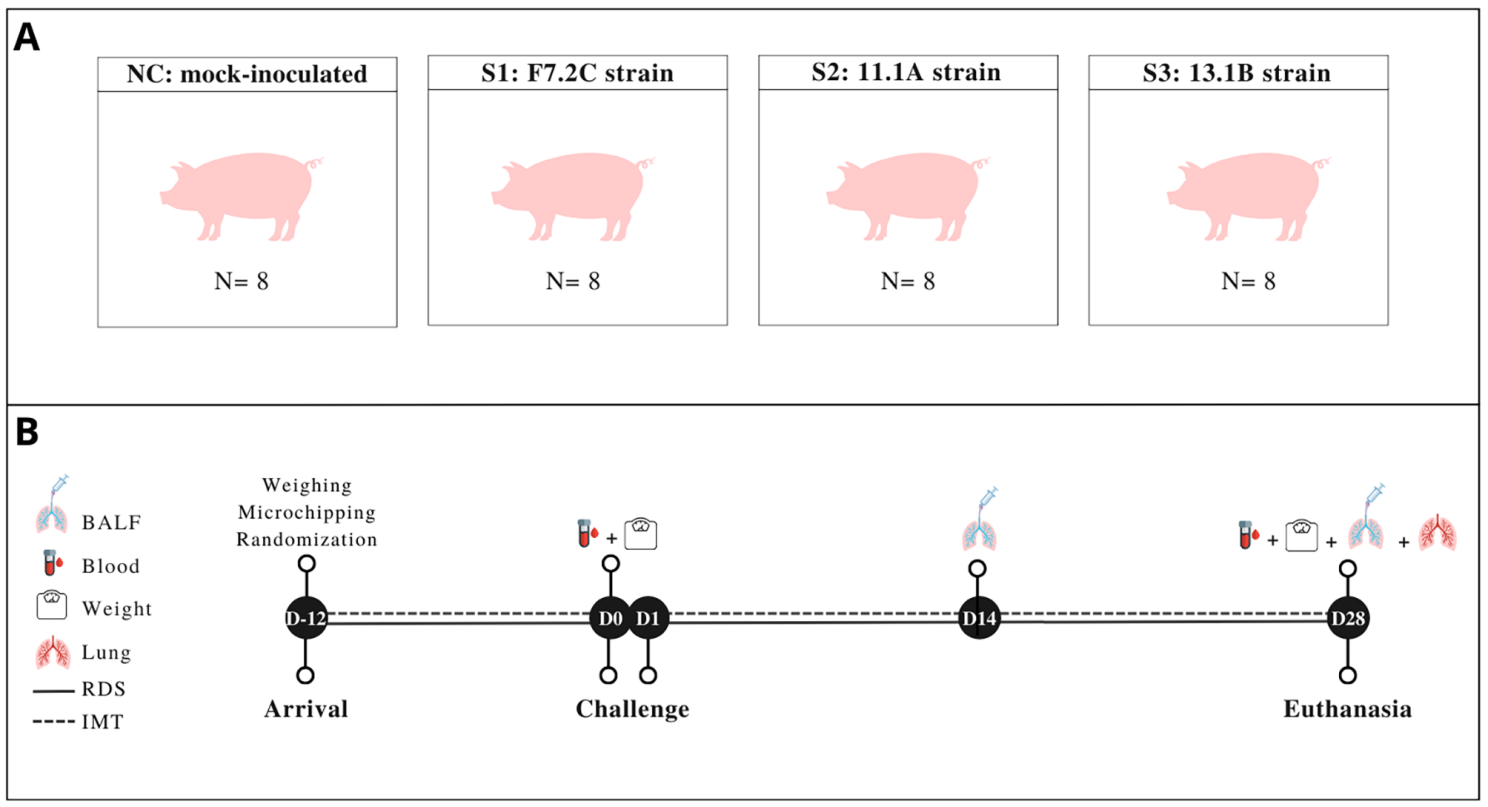
Schematic overview of the study design. **A**) Thirty-two *M. hyopneumoniae*-free piglets were randomly allocated to the four groups. **B**) Overview of the timeline and sampling events. NC: negative control group inoculated with sterile medium on D0 and D1; S1: group infected with the *M. hyopneumoniae* strain F7.2 C (D0) and F1.12 A (D1); S2: group infected with the recently isolated *M. hyopneumoniae* strain 11.1 A (D0) and F1.12 A (D1); S3: group infected with the recently isolated *M. hyopneumoniae* strain 13.1B (D0) and F1.12 A (D1); BALF: broncho-alveolar lavage fluid; RDS: respiratory disease score; IMT: intramuscular temperature, D: day

The piglets were transported to the research facilities of the Faculty of Veterinary Medicine, at Ghent University, when they were 28 days old (D-12). Upon arrival, the piglets were weighted, and identified with numbered ear tags, and microchips (2.12 × 13 mm) with Bio-Thermo technology were implanted intramuscularly (LifeChip^®^; Allflex, Belgium) for temperature measurement. Then, the animals were randomly allocated to four different groups (*n* = 8/group) (Fig. 3), and able to acclimatize for 12 days.

Each group was housed in one of the four separate, but similar HEPA-filtered compartments, and with free access to drinking water and feed. The animals were fed with a commercial feed free from antibiotics. In addition, feed and water management, environmental conditions, and other factors such as flooring, heating, the number and location of toys, and feeders were consistent among the different compartments/groups. Each compartment was equipped with a Healthy Climate Monitor (HCM; Healthy Climate Solutions, The Netherlands), a device used to monitor the movement of the animals, sound levels, and environmental parameters in pig farms. Details about the HCM are described under the section “Assessment of movement, sound, and environmental conditions”.

If pigs showed clinical signs of post-weaning diarrhea, they were treated orally with colistin (Colivet^®^ quick-pump, Prodivet, Belgium), and in case of lameness, they were treated intramuscularly with amoxicillin (Duphamox LA, Zoetis, Belgium) and meloxicam (Melovem^®^, Dopharma, Belgium), following the leaflet dosage.

### Isolates

The F7.2 C strain (GenBank accession #NC_007295) and F1.12 A strain (GenBank accession #KY264124.1) were isolated in the year 2000 and were characterized as highly and low virulent strains [7], respectively. On the other hand, the strains 11.1 A (GenBank accession # CP098241) and 13.1B (GenBank accession # CP098240) were recently isolated (2021) from pigs with *Mycoplasma*-like lung lesions at slaughter. Their virulence has not yet been studied.

The new strains were isolated from two different fattening herds in Belgium, and whole-genome sequencing (WGS), on a MinION flow cell (Oxford Nanopore Technologies), confirmed that they belong to the *M. hyopneumoniae* species and were distinctly related to the F7.2 C strain. The pairwise identity ranged from 97.92 to 99.45% (BLASTN, v2.10.11; data not published). Last, the genome of the isolates 11.1 A and 13.1B is 0.95Mbp and 0.98Mbp, respectively (data not published).

### Inoculum preparation and challenge

One milliliter of each *M. hyopneumoniae* isolate (13.1B, 11.1 A, F7.2 C, or F1.12 A) was inoculated in a flask (one for each strain) containing 400 ml of Friis medium [45, 46], which was maintained in an incubator at 37 °C until ATP values [46] were greater than 430 pmol ATP/ml. The concentration of *M. hyopneumoniae* was determined by successive dilutions of the culture in Friis medium as described elsewhere [46]. Then, the inoculum was aliquoted into 50 ml sterile falcons and stored at -80 °C until use. Sterility tests were performed for all flasks on blood agar and McConkey media, and left in an incubator at 37 °C for two days. The sterility test turned out to be negative for all cultures/flasks.

All pigs were inoculated at 40 and 41 days of age (D0 and D1). On the first inoculation day (D0), piglets (*n* = 8) in each infected group received one of the three different *M. hyopneumoniae* strains namely F7.2 C (S1), 11.1 A (S2), or 13.1B (S3). The following day (D1), piglets in the three infected groups were additionally inoculated with a low virulent strain (F1.12 A). The negative control group was inoculated with sterile Friis medium at both time points (D0 and D1).

Before inoculation, animals were anesthetized with 0.22 mL/kg body weight of a mixture of Zoletil 100^®^ (Virbac, Belgium) and Xyl-M^®^ 2% (VMD, Belgium), which was given intramuscularly according to label instructions. The challenge was performed by endotracheal inoculation of 7 ml of each *M. hyopneumoniae* strain containing 10^8^ CCU/ml [46] or sterile Friis medium.

### Assessment of movement, sound, and environmental conditions

The HCM (Healthy Climate Solutions, The Netherlands) is a tool designed for monitoring the indoor farm climate. The device measures room temperature, relative humidity, carbon dioxide (CO_2_), ammonia (NH_3_), dust particles (10 µg/m^3^ and 1 µg/m^3^), and air pressure. In addition, it captures high-definition photos to assess movement and includes an optional microphone for measuring the noise levels (sound spectrum from 172 Hz to 10 kHz). The device transmits the data, once per minute over a 4G connection, to the Healthy Climate Monitor app, which can then be used by the researcher. The percentage of movement was calculated automatically by an algorithm (Healthy Climate Solutions Api, version 2.7.3), which compares the changes in pixels between images (every 5 min), with a resolution of 1640 × 1232px.

In each room, an HCM device was installed on the ceiling in the middle, away from the air inlet, and approximately two meters high from the floor, allowing a complete view over the pen and the animals (Additional file 8). The movement of the piglets was continuously measured by the HCM from D-11 to D28. However, only measurements from 11 am to 5 pm were used to avoid possible interference with the handling of the animals in the morning, and bias from technical issues during the nighttime (like not capturing pictures or sudden changes in brightness), which interfered with the pixels count, and therefore the percentage of movement. Lastly, the percentage of movement was calculated as the daily mean of the measurements taken within the 6-hour period.

### Assessment of clinical signs, body temperature, and average daily weight gain (ADWG)

During the study, the animals were observed daily, by the same person, for at least 20 min in the morning (between 8-10am). The severity of coughing was measured using a Respiratory Disease Score (RDS) [47], which ranges from 0 to 6, where: 0 = no coughing, 1 = mild coughing after an encouraged move, 2 = mild coughing in rest, 3 = moderate coughing after an encouraged move, 4 = moderate coughing in rest, 5 severe coughing after an encouraged move, 6 = severe coughing in rest. Other possible clinical findings, such as loss of appetite, diarrhea, dyspnea, and lameness were also recorded.

Two different methods were used to evaluate the body temperature of the piglets: manually taken rectal temperature (RT) and with a transponder-read thermal microchip (LifeChip^®^, Allflex, Belgium). The microchip was implanted intramuscularly, on the right side of the neck (Additional file 9), according to the instructions provided by the manufacturer. The individual intramuscular temperature was assessed daily in the morning (between 8.00 and 10.00) from D-11 to D28 by reading the transponder with a portable stick reader RS 420 (Allflex, Belgium). The RT was measured daily from D-7 to D-3 (between 8.00 and 10.00), and after the IMT measurement, with a digital thermometer. Last, to investigate possible migration, the location of the microchips was checked in 20% of the animals at necropsy.

All piglets were weighed upon arrival (D-12), before inoculation (D0), and at euthanasia (D28) to evaluate the average daily weight gain. The ADWG was calculated by subtracting the weight at D-12 or D0 from the final weight (D28) and dividing the difference by the number of days between the time points.

### Blood and broncho-alveolar lavage fluid (BALF) sampling

Blood samples were collected just before inoculation (D0) and at euthanasia (D28) by puncturing the jugular vein, using sterile disposable needles and vacutainer tubes with clot activator (Vacutainer - BD, USA). Blood samples were centrifuged at 1500x*g* for 10 min and the serum was aliquoted into sterile microtubes and stored at -20 °C until use.

The BALF samples were collected two weeks after inoculation (D14) and at euthanasia (D28). At D14, the animals were restrained using a snare and a catheter (Portex^®^ Dog Catheter with Female Luer Mount, United Kingdom) was inserted into the trachea. Then, 20 mL sterile PBS was flushed into the lung and immediately aspirated back. At D28, BALF samples were collected after euthanasia by flushing the head bronchus of the right lung with 20 mL sterile PBS, followed by immediate aspiration of the fluid. All BALF samples were aliquoted into sterile microtubes and stored at -80 °C until further testing by PCR.

### Macroscopic and microscopic lung lesion evaluation

After the lungs were removed from the cadaver at necropsy (D28), the EP-like lung lesion or macroscopic lung consolidated lesions (MLCL) were scored according to Hannan et al. [48]. The score ranges from 0 (no EP-like lesions) to 35 (entire lung affected by EP-like lesions).

For the microscopic lung lesion (MLL) evaluation, samples from the left lung (apical, cardiac, and diaphragmatic lobes) were collected [48], fixed in 10% neutral buffered formalin for 48 h, and processed for hematoxylin and eosin staining. If EP-like lesions were present, the sample was collected from the transition area of the lesion. The microscopic slides were digitalized using a Nanozoomer NDP slide scanner (Hamamatsu Photonics, Japan) and its viewing platform (NDP.View2). To get the average lesion score, the degree of peribronchiolar and perivascular lymphohistiocytic infiltration and nodule formation (cuffing) was assessed on 10 microscopic fields (10x magnification) using a previously described scoring system [7, 49]. Briefly, the score ranges from 1 to 5, with 1 = limited infiltration of macrophages and lymphocytes around bronchioles, with airways and alveolar spaces free of cellular exudates; 2 = light to moderate infiltrates with mild diffuse cellular exudates into airways; 3, 4, and 5 (mild, moderate and severe, respectively) lesions characteristic of broncho-interstitial pneumonia, centered around bronchioles but extending to the interstitium, with lymph follicular infiltration and mixed inflammatory cell exudates (Additional file 10). The percentage of lung area occupied by air (% of air) in the lung was determined by analyzing the same 10 microscopic fields per lobe with the ImageJ program (Bethesda Softworks, USA), as reported elsewhere [17, 37].

### Routine bacteriological culture

Ten microliters of each BALF sample collected at euthanasia (D28) were inoculated on Columbia blood agar plates with 5% sheep blood (Oxoid, United Kingdom) with a *Staphylococcus pseudintermedius* streak, as reported previously [50]. All plates were incubated for 48 h at 37 °C in a 5% CO_2_-enriched environment to isolate other bacteria, especially respiratory pathogens. Then, the different colonies were identified at the species level (score value > 2.000) using matrix-assisted laser desorption/ionization time-of-flight mass spectrometry (MALDI-TOF; Bruker Daltonics, Germany) as previously described [17, 51, 52].

### Anti-M. hyopneumoniae antibody detection

To detect specific serum antibodies at D0 and D28, the commercial kit *M.hyo Ab test* (Idexx, USA) was used, following the manufacturer’s instructions. Absorbance values were read in a spectrophotometer at 650 nm wavelength. All samples with S∕P values higher than 0.3 were considered positive for anti-*M. hyopneumoniae* antibody levels [35].

### DNA extraction and digital PCR (dPCR) for M. hyopneumoniae

A total of 200 µL of each BALF sample was used for DNA extraction. The DNeasy Blood & Tissue kit (QIAGEN, Belgium) was used according to the manufacturer’s protocol. Then, digital droplet PCR (dPCR) was performed as described previously [17]. The assay is based on the following primers and probe: FW-primer 5’-GTCAAAGTCAAAGTCAGCAAAC; RV-primer 5’-AGCTGTTCAAATGCTTGTCC; and the probe 5’-Cy5ACCAGTTTC-TAO-CACTTCATCGCCTCA-IAbRQSp, which target the *p*102 adhesin gene [53]. The assay was performed with the droplet-based Naica System (Stilla Technologies, France) using 16 reaction cavity Naica Opal chips (Stilla Technologies, France). Briefly, the dPCR mix (8 µL) containing 1x PerfeCTa^®^ Multiplex qPCR Toughmix^®^ (VWR International BVBA, Belgium), 0.25 µmol/L of the probe, 0.5 µmol/L of each primer, 0.1 µmol/L fluorescein (Merck, Germany), 0.4 µL Fast Digest EcoRi (Thermo Fisher Scientific, USA) and 1.6 µL sample DNA. Highly concentrated samples were diluted using the UltraPure™ Salmon Sperm DNA Solution (10 µg/mL, Thermo Fisher Scientific, USA). For each run, at least two non-template controls (NTC) were included. The thermal cycling was performed on the Geode device (Stilla Technologies, France), where initial denaturation (10 min at 95 °C) was followed by 40 amplification cycles of denaturation for 15 s at 95 °C and annealing/elongation for 30 s at 60 °C. Fluorescence reading was done using the Naica Prism3 System (Stilla Technologies, France). The threshold value was based on the NTC samples, as described elsewhere [17].

### Statistical analyses

The results were analyzed using IBM SPSS^®^ Statistics Version 28 (IBM, Chicago, USA). For all models, the assumption of normality was tested by examining histograms, and the homogeneity of variance of the final models was tested by examining histograms and the QQ plot of the residuals. The assumption of homogeneity of residuals was checked before using parametric tests/models (e.g. Bland Altman test) by the Shapiro-Wilk test. For the mixed models used in this study, the assumption of homogeneity of residuals was checked by plotting residuals versus fitted values.

To evaluate the data statistically, all animals in the infected groups (S1, S2, and S3) were pooled and considered as one group, the *M. hyopneumoniae*-infected group, whereas the NC remained as the negative control group. The linear mixed model with repeated measurements was used to evaluate the movement data, in which movement was the dependent variable and infection status was the independent variable. The Generalized Estimating Equations model was used to analyze the RDS data, where the infected groups were pooled and considered as one group (independent variable).

The correlations between the main clinical parameters (MLCL, MLL, IMT, RT, RDS, *M. hyopneumoniae*-DNA load, anti-*M. hyopneumoniae* antibody levels, and percentage of air) were evaluated by the Spearman’s method. The association between intramuscular and rectal temperature was assessed by Bland Altman graphs and non-parametric tests. Statistical results were considered significant when *p* ≤ 0.05.

### Electronic supplementary material

Below is the link to the electronic supplementary material.

Supplementary Material 1

## Data Availability

No datasets were generated or analysed during the current study.
