# Supplementary material for: Rationalizing the use of common parameters and technological tools to follow up Mycoplasma hyopneumoniae infections in pigs
Source: Porcine Health Manag. 2024 Aug 23;10:31. doi: 10.1186/s40813-024-00381-x (PMC11342468; doi:10.1186/s40813-024-00381-x)
Supplement: Supplementary file 1 — Supplementary Material 1 [file 40813_2024_381_MOESM1_ESM.docx]

**Additional Files**


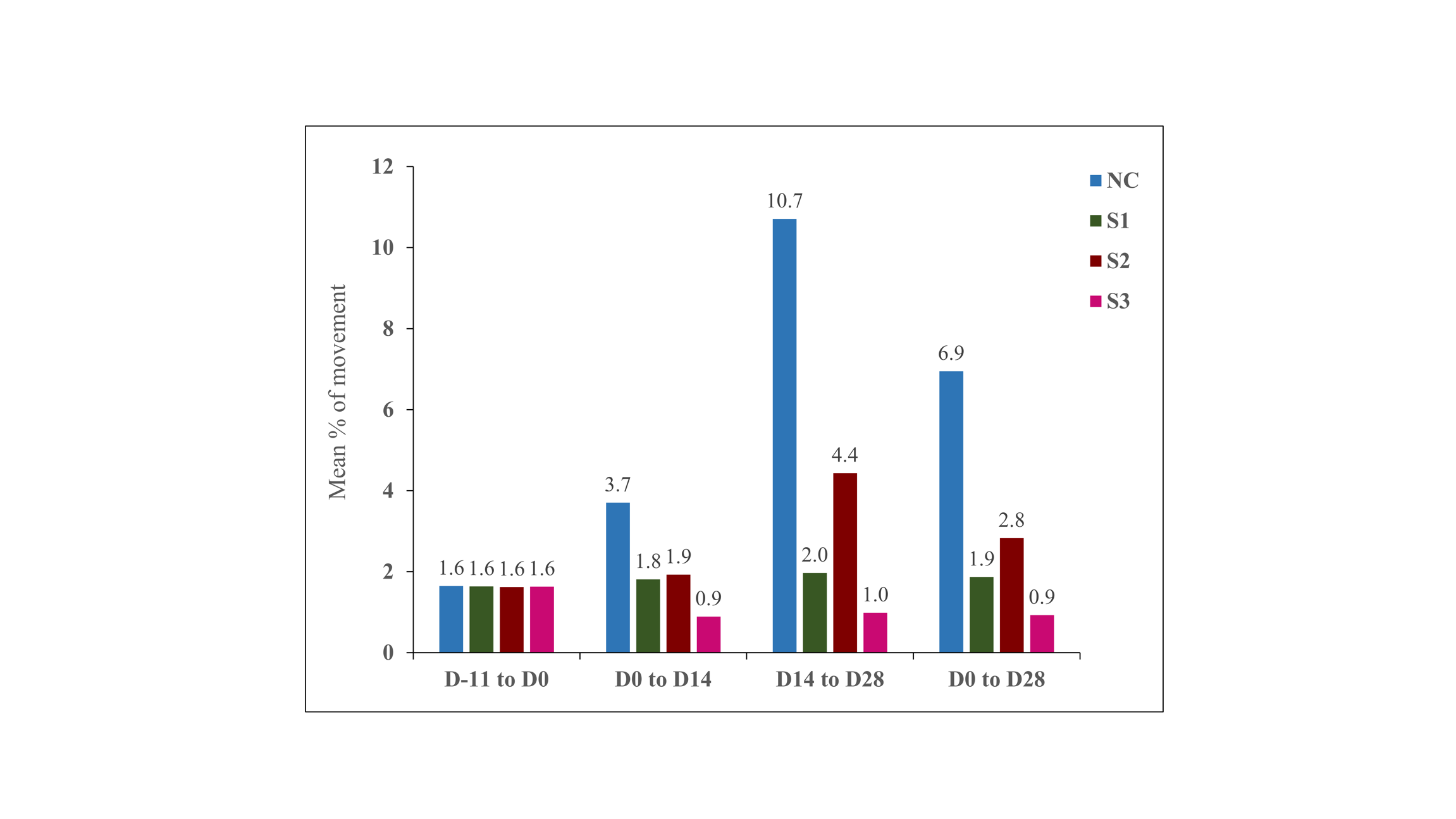
**Additional file 1**. **Figure.** Mean percentage of movement (y axis) in the experimental groups according to the study period (x axis). NC: Negative control group; S1: group infected with the *M. hyopneumoniae* strain F7.2C; S2: group infected with *M. hyopneumoniae* strain 11.1A; S3: group infected with *M. hyopneumoniae* strain 13.1B; D: day.

**Additional file 2. Table.** Detailed values on the main environmental parameters measured by the Healthy Climate Monitor throughout the study (D-12 to D28). NC: Negative control group; S1: group infected with the F7.2C *M. hyopneumoniae* strain; S2: group infected with the recently isolated 11.1A *M. hyopneumoniae* strain; S3: group infected with the recently isolated 13.1B *M. hyopneumoniae* strain.

| **Parameter** | **Group** | **Median** | **Min.** | **Max** |
| --- | --- | --- | --- | --- |
| Sound 602 Hz (dB) | NC | 42.20 | 39.00 | 51.30 |
|  | S1 | 39.60 | 39.00 | 48.80 |
|  | S2 | 39.30 | 39.00 | 45.70 |
|  | S3 | 39.50 | 39.00 | 49.70 |
| CO_2_ (ppm) | NC | 571.60 | 347.00 | 1030.90 |
|  | S1 | 618.80 | 522.20 | 1192.60 |
|  | S2 | 631.55 | 512.90 | 867.60 |
|  | S3 | 717.40 | 617.00 | 950.60 |
| NH_3_ (ppm) | NC | 0.00 | 0.00 | 3.30 |
|  | S1 | 0.00 | 0.00 | 2.20 |
|  | S2 | 0.00 | 0.00 | 4.50 |
|  | S3 | 0.00 | 0.00 | 3.30 |
| Ambient temperature (°C) | NC | 25.00 | 21.20 | 26.60 |
|  | S1 | 24.00 | 19.80 | 25.60 |
|  | S2 | 24.40 | 20.60 | 28.30 |
|  | S3 | 24.00 | 21.10 | 27.50 |
| Relative humidity (%) | NC | 34.90 | 24.30 | 52.40 |
|  | S1 | 34.60 | 24.60 | 53.20 |
|  | S2 | 38.80 | 28.00 | 57.20 |
|  | S3 | 29.40 | 14.10 | 46.90 |
| Air pressure (hPa) | NC | 1018.70 | 983.90 | 1031.40 |
|  | S1 | 1019.00 | 983.90 | 1031.60 |
|  | S2 | 1019.60 | 984.10 | 1031.70 |
|  | S3 | 1019.10 | 984.20 | 1031.80 |
| PM 10 (µg/m3) | NC | 44.60 | 37.50 | 203.20 |
|  | S1 | 2.00 | 1.00 | 42.00 |
|  | S2 | 1.00 | 1.00 | 11.50 |
|  | S3 | 1.00 | 1.00 | 13.80 |
| PM 1 (µg/m3) | NC | 44.40 | 37.50 | 202.90 |
|  | S1 | 1.10 | 0.00 | 41.00 |
|  | S2 | 0.85 | 0.00 | 3.00 |
|  | S3 | 1.00 | 0.00 | 7.60 |

dB= decibels; ppm= parts per million; hPa= hectopascal; PM: particle matter


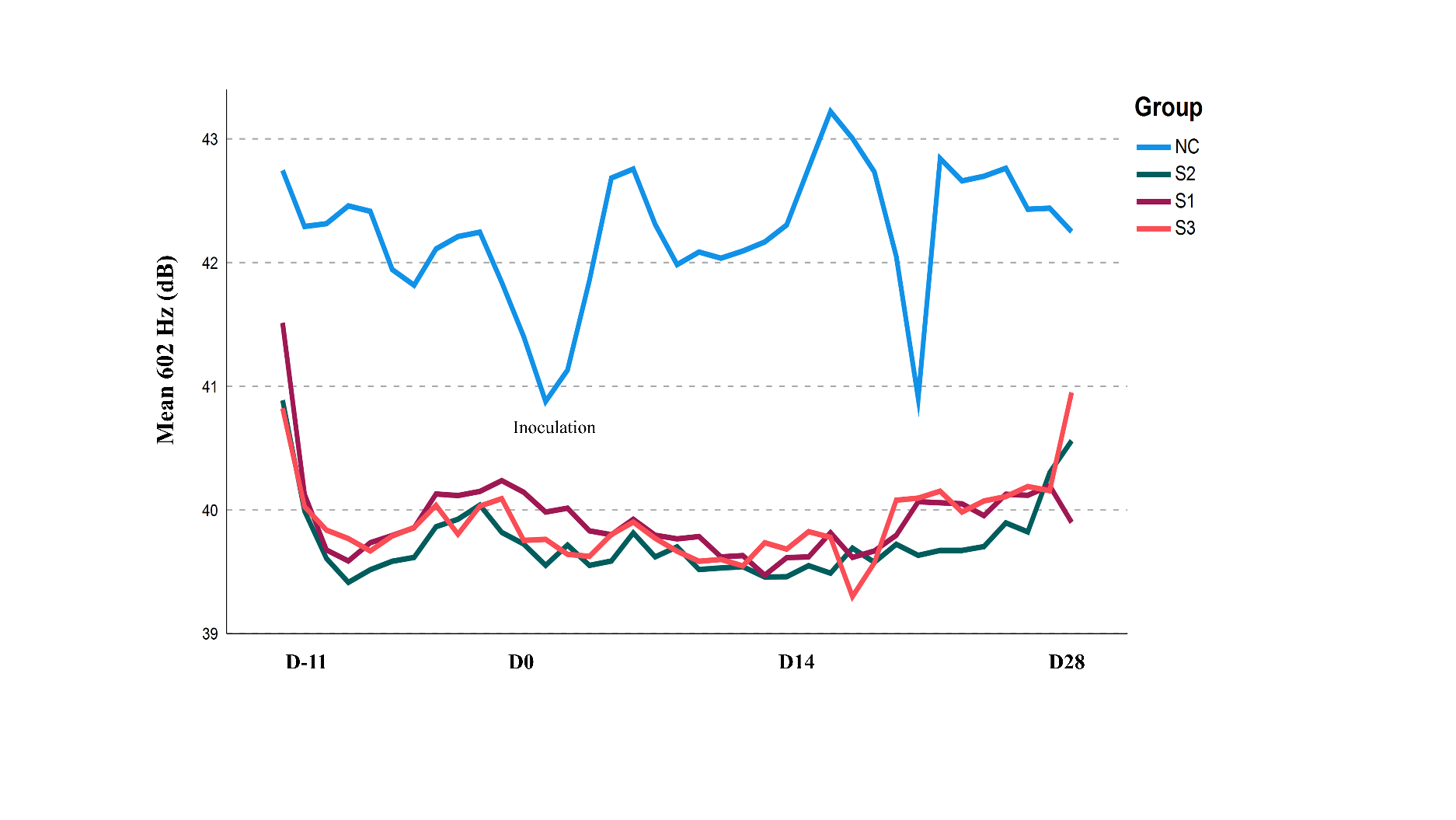


**Additional file 3.** **Figure.** Mean values of the 602 Hz frequency by group, where the daily average was calculated based on several measurements in the 24-hour period from D-11 to D28. Overall, higher values were observed in the negative control group. NC: Negative control group; S1: group infected with *M. hyopneumoniae* strain F7.2C; S2: group infected with *M. hyopneumoniae* strain 11.1A; S3: group infected with *M. hyopneumoniae* strain 13.1B. D: day.


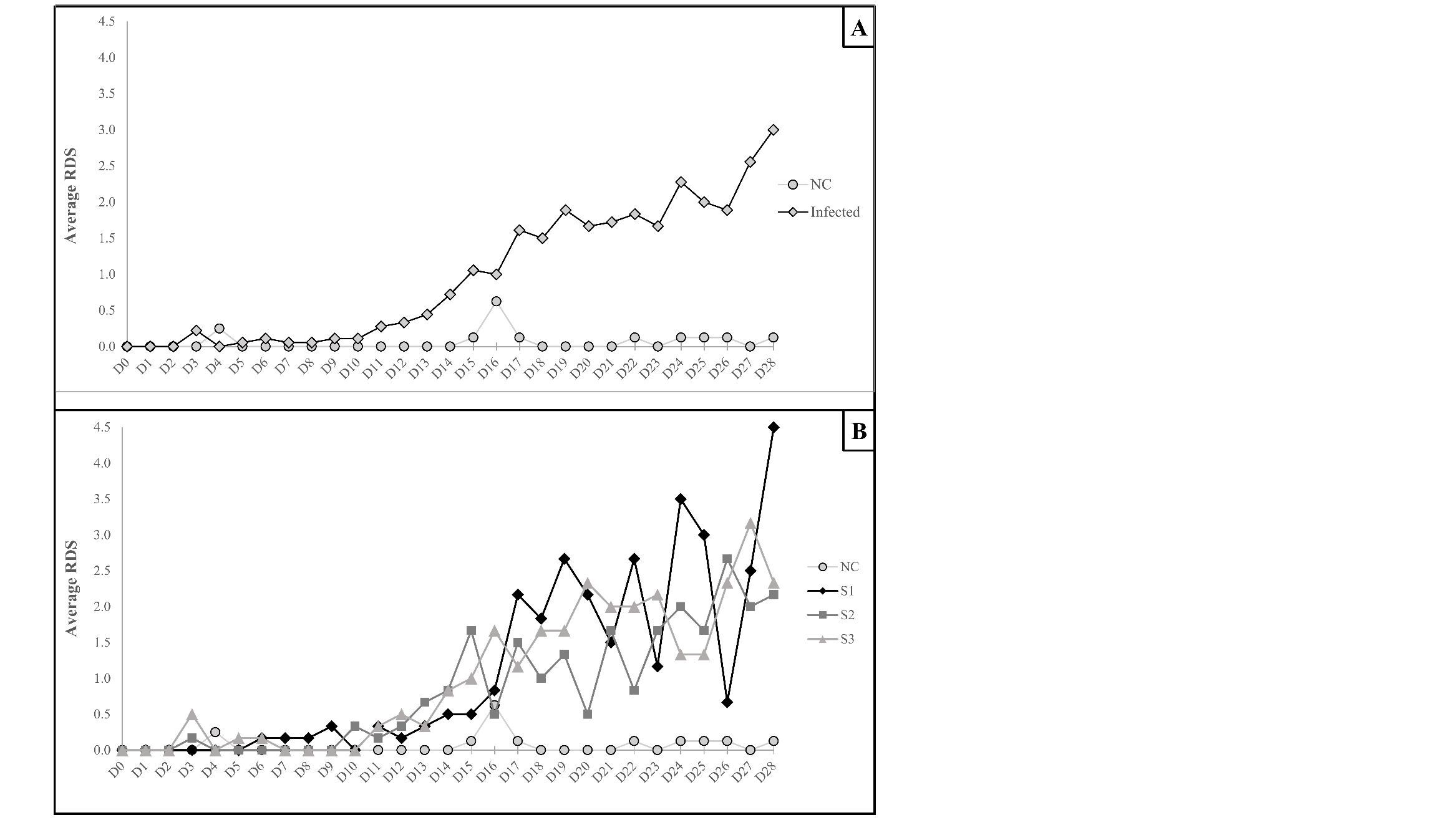


**Additional file 4.** **Figure.** Average Respiratory Disease Score (RDS) data by treatment (A) and by the individual groups (B). NC: Negative control group; S1: group infected with the F7.2C *M. hyopneumoniae* strain; S2: group infected with the recently isolated 11.1A *M. hyopneumoniae* strain; S3: group infected with the recently isolated 13.1B *M. hyopneumoniae* strain; D: day.


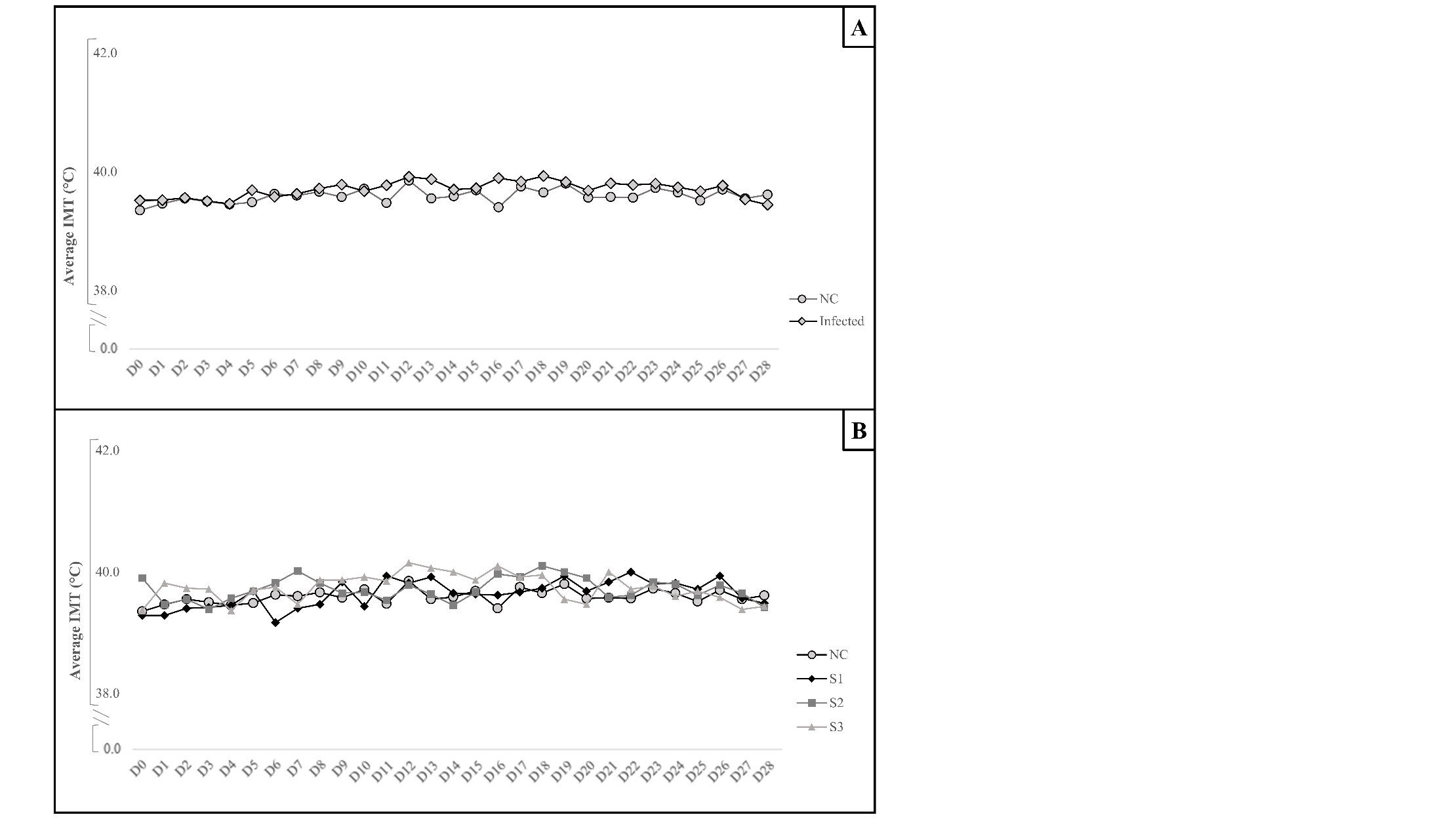


**Additional file 5.** **Figure.** Average intramuscular temperature (IMT) data by treatment (A) and by the individual groups (B). NC: Negative control group; S1: group infected with the F7.2C *M. hyopneumoniae* strain; S2: group infected with the recently isolated 11.1A *M. hyopneumoniae* strain; S3: group infected with the recently isolated 13.1B *M. hyopneumoniae* strain; IMT: intramuscular temperature, D: day.

**
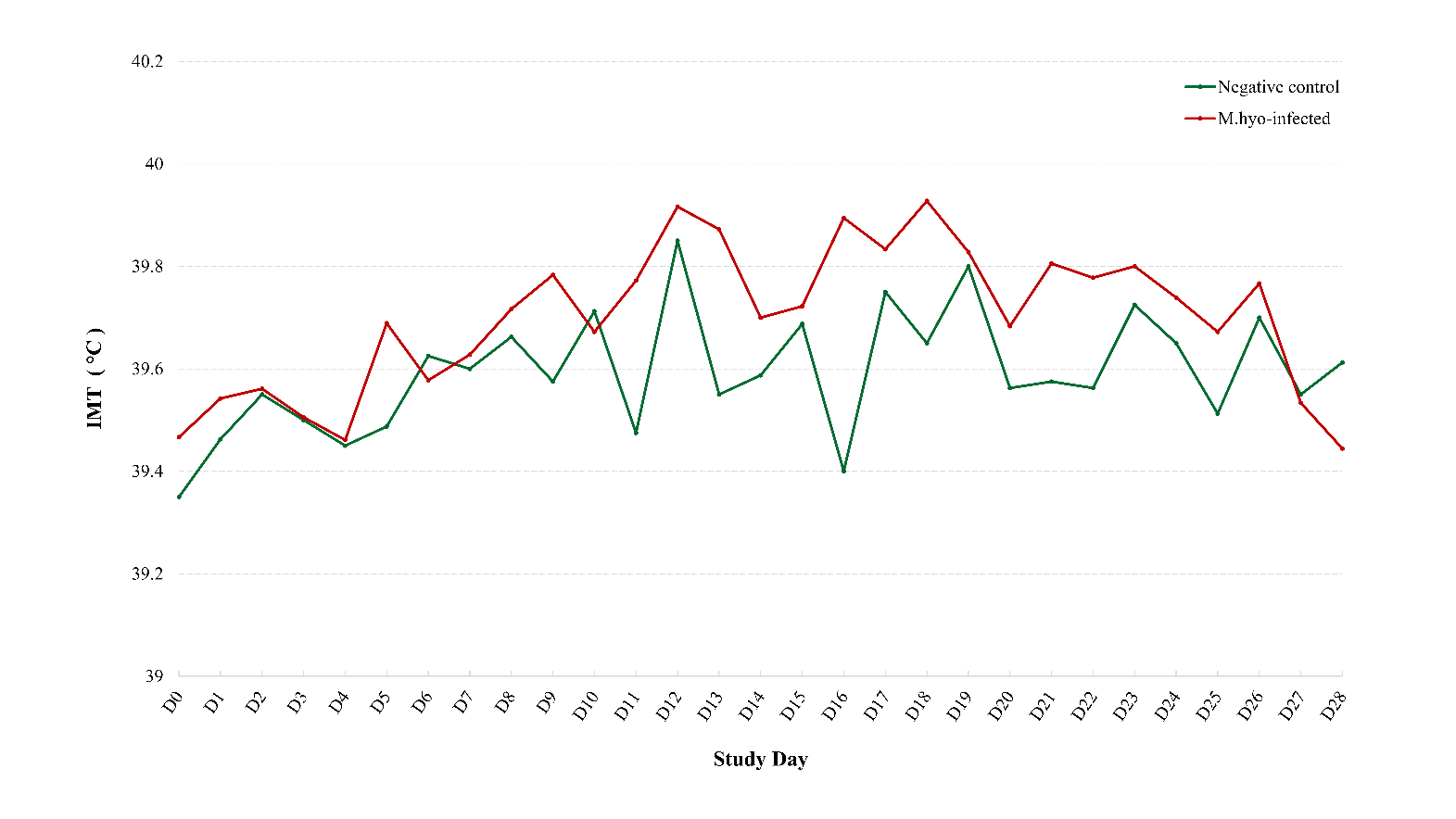
**

**Additional file 6.** **Figure.** Average post-inoculation (D0-D28) intramuscular temperature (IMT) by treatment groups (Negative control and *M. hyopneumoniae*-infected group (*M.hyo*-infected). D: day.

**Additional File 7.** **Table**. Mean and standard deviation (sd) of the main clinical parameters evaluated by group. RDS: respiratory disease score; IMT: intramuscular body temperature; ADWG: average daily weight gain; MLCL: macroscopic lung consolidated lesion; MLL: microscopic lung lesion; D: day.

| **Parameter** |  | **Study day** |  | **NC** | |  | **S1** | |  | **S2** | |  | **S3** | |
| --- | --- | --- | --- | --- | --- | --- | --- | --- | --- | --- | --- | --- | --- | --- |
|  |  |  |  | mean | sd |  | mean | sd |  | mean | sd |  | mean | sd |
| *RDS* |  | D-11 to D0 |  | 0.0 | 0.0 |  | 0.0 | 0.0 |  | 0.0 | 0.0 |  | 0.0 | 0.0 |
|  |  | D-11 to D28 |  | 0.0 | 0.1 |  | 0.8 | 0.4 |  | 0.6 | 0.4 |  | 0.7 | 0.3 |
|  |  | D0 to D28 |  | 0.1 | 0.2 |  | 1.1 | 0.9 |  | 0.8 | 0.9 |  | 1.0 | 0.7 |
|  |  | D14 to D28 |  | 0.1 | 0.1 |  | 2.0 | 0.5 |  | 1.5 | 0.5 |  | 1.8 | 0.4 |
| *IMT in °C* |  | D0 to D5 |  | 39.5 | 0.2 |  | 39.4 | 0.2 |  | 39.6 | 0.2 |  | 39.6 | 0.3 |
|  |  | D0 to D10 |  | 39.6 | 0.2 |  | 39.4 | 0.2 |  | 39.7 | 0.2 |  | 39.7 | 0.3 |
|  |  | D0 to D28 |  | 39.6 | 0.2 |  | 39.6 | 0.1 |  | 39.7 | 0.2 |  | 39.7 | 0.3 |
| *ADWG in g/pig/day* |  | D-12 to D0 |  | 114 | 48 |  | 142 | 58 |  | 119 | 28 |  | 80 | 57 |
|  |  | D-12 to D28 |  | 328 | 90 |  | 351 | 97 |  | 414 | 42 |  | 322 | 110 |
|  |  | D0 to D28 |  | 419 | 124 |  | 440 | 132 |  | 540 | 57 |  | 426 | 141 |
| *M. hyo-antibody values* |  | D0 |  | 0.0 | 0.0 |  | 0.0 | 0.0 |  | 0.0 | 0.0 |  | 0.0 | 0.0 |
|  |  | D28 |  | 0.0 | 0.0 |  | 0.9 | 0.2 |  | 0.6 | 0.3 |  | 0.6 | 0.3 |
| *M. hyo-DNA load (log10)* |  | D14 |  | 0.0 | 0.0 |  | 4.0 | 0.8 |  | 3.5 | 0.2 |  | 4.1 | 0.4 |
|  |  | D28 |  | 0.0 | 0.0 |  | 4.7 | 0.3 |  | 4.3 | 0.3 |  | 4.3 | 0.4 |
| *MLCL score* |  | D28 |  | 0.6 | 1.3 |  | 3.9 | 1.8 |  | 4.4 | 2.5 |  | 4.7 | 2.9 |
| *MLL score* |  | D28 |  | 2.2 | 0.2 |  | 2.4 | 0.2 |  | 2.7 | 0.4 |  | 2.8 | 0.4 |
| *Percentage of air in lung tissue* |  | D28 |  | 37.3 | 4.5 |  | 37.4 | 5.9 |  | 33.4 | 5.7 |  | 32.9 | 6.8 |

NC: Negative control group; S1: group infected with the F7.2C *M. hyopneumoniae* strain; S2: group infected with the recently isolated 11.1A *M. hyopneumoniae* strain; S3: group infected with the recently isolated 13.1B *M. hyopneumoniae* strain; D: day.


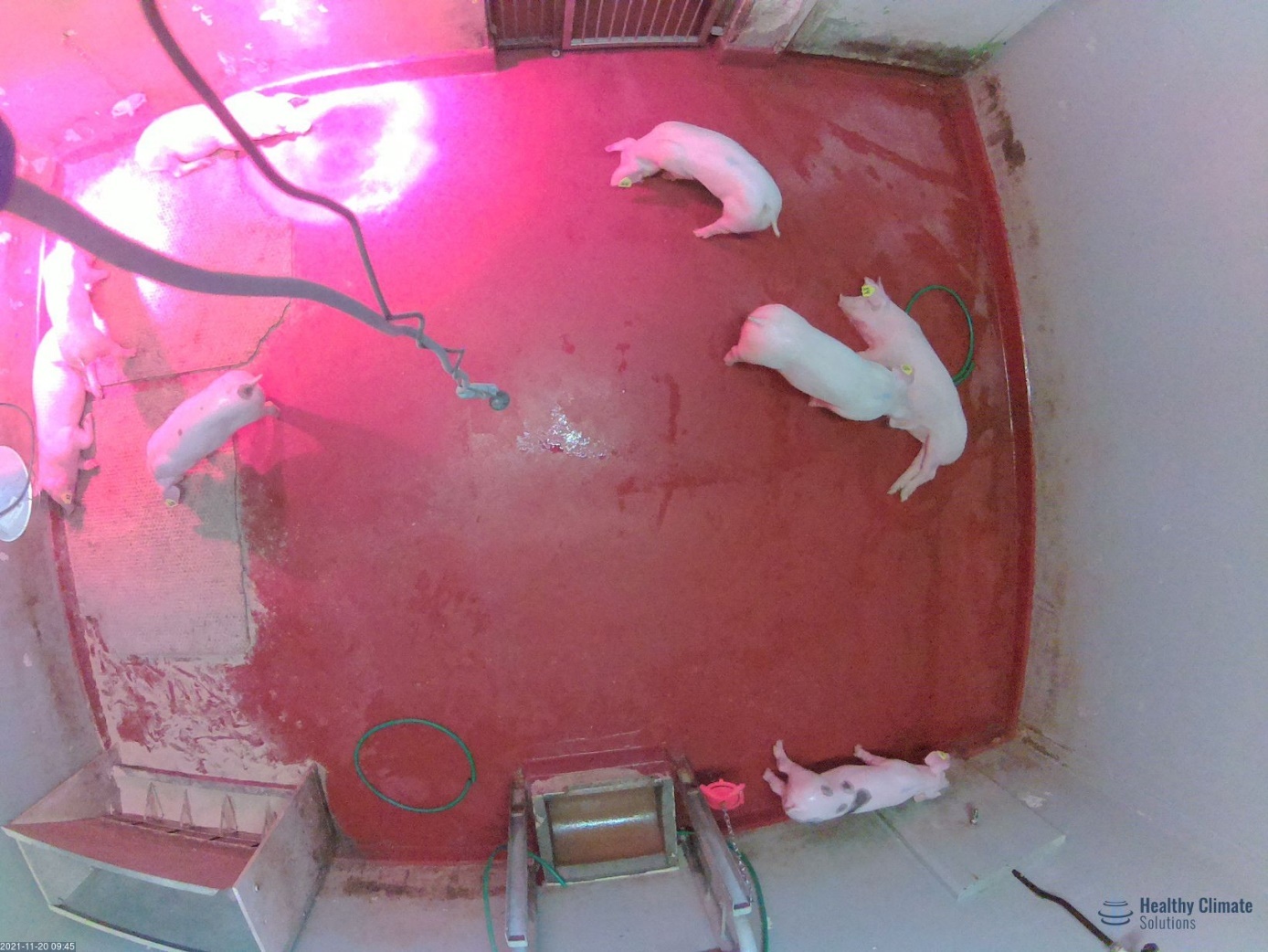


**Additional File 8**. **Picture.** Image obtained from the Healthy Climate Monitor showing a complete view of the negative control room.


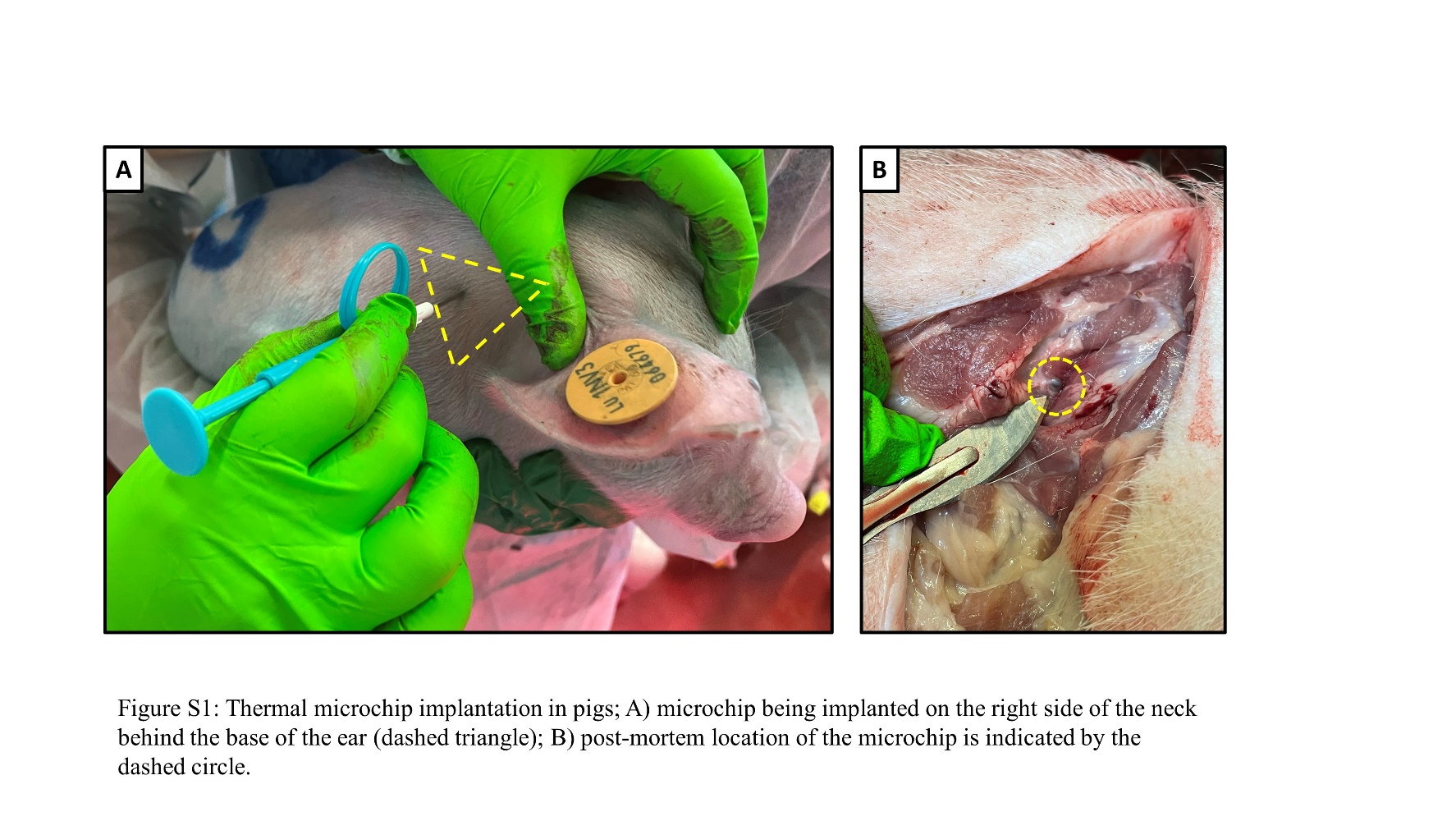


**Additional File 9**. **Picture.** Thermal microchip implantation in pigs; A) microchip being implanted on the right side of the neck behind the base of the ear (dashed triangle); B) post-mortem location of the microchip is indicated by the dashed circle.


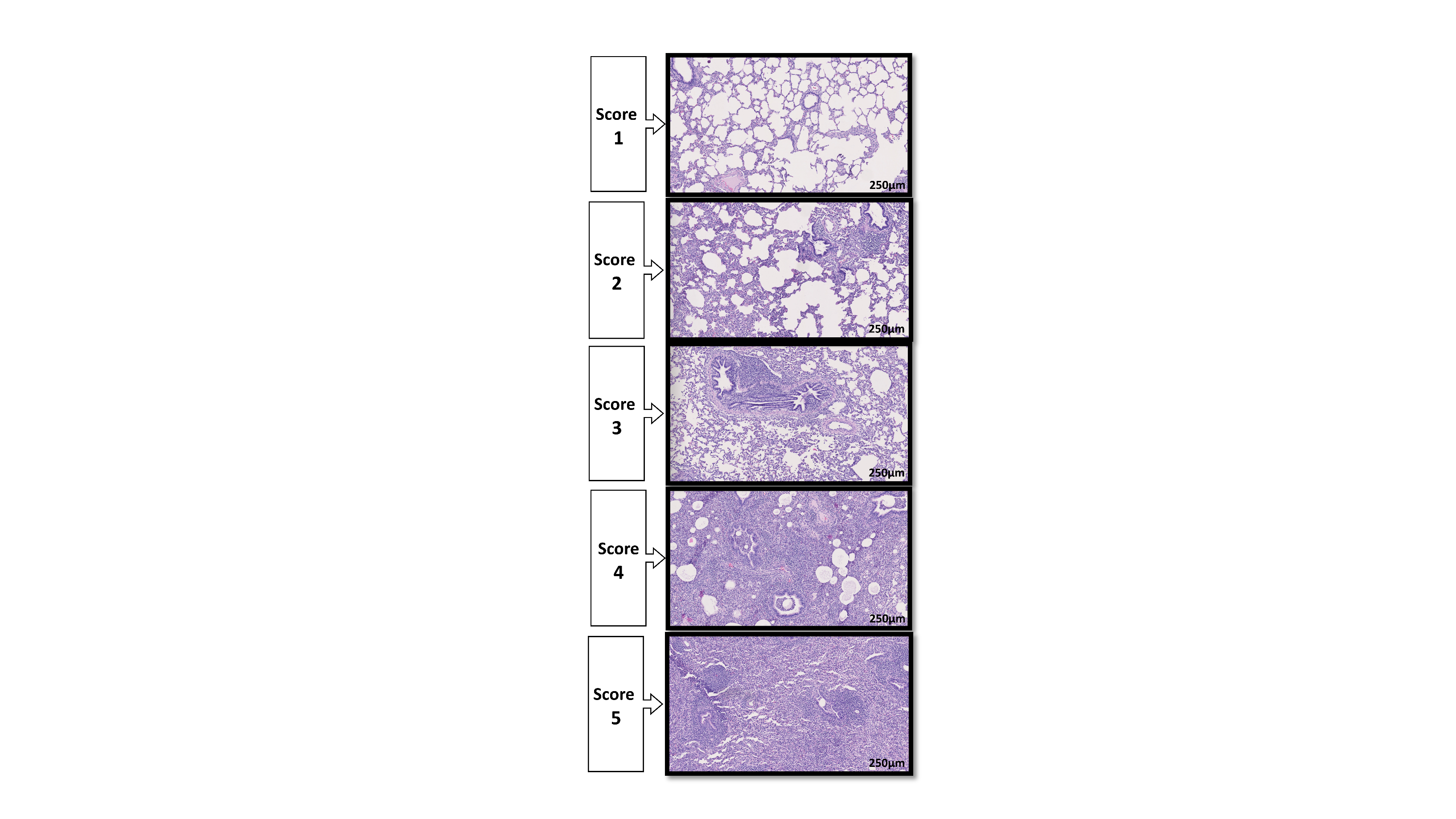


**Additional File 10**. **Picture.** Microscopic lung lesion scoring adapted from Morris et al. (Morris et al. 1995) and Vicca et al. (Vicca et al. 2003). Score 1: limited infiltration of macrophages and lymphocytes around bronchioles, with airways and alveolar spaces free of cellular exudates; Score 2 = light to moderate infiltrates with mild diffuse cellular exudates into airways; Scores 3, 4, and 5 (mild, moderate and severe, respectively) lesions characteristic of broncho-interstitial pneumonia, centered around bronchioles but extending to the interstitium, with lymph follicular infiltration and mixed inflammatory cell exudates. The pictures as shown as 10X magnification.

Morris, CR, IA Gardner, SK Hietala, and TE Carpenter. 1995. 'Enzootic pneumonia: comparison of cough and lung lesions as predictors of weight gain in swine', *Canadian Journal of Veterinary Research*, 59: 197-204.

Vicca, J., T. Stakenborg, D. Maes, P. Butaye, J. Peeters, A. de Kruif, and F. Haesebrouck. 2003. 'Evaluation of virulence of Mycoplasma hyopneumoniae field isolates', *Veterinary microbiology*, 97: 177-90.
